# CFD Computation of Flow Fractional Reserve (FFR) in Coronary Artery Trees Using a Novel Physiologically Based Algorithm (PBA) Under 3D Steady and Pulsatile Flow Conditions

**DOI:** 10.3390/bioengineering10030309

**Published:** 2023-02-28

**Authors:** Nursultan Alzhanov, Eddie Y. K. Ng, Xiaohui Su, Yong Zhao

**Affiliations:** 1 Mechanical and Aerospace Engineering Department, School of Engineering, Nazarbayev University, Asana 010000, Kazakhstan; 2 School of Mechanical and Aerospace Engineering, Nanyang Technological University, Singapore 639798, Singapore; 3 School of Mechanical Engineering, Dalian University of Technology, Dalian 116024, China

**Keywords:** FFR, blood flow simulation, coronal stenosis, coronary computed tomography angiography (CCTA), OpenFOAM

## Abstract

A novel physiologically based algorithm (PBA) for the computation of fractional flow reserve (FFR) in coronary artery trees (CATs) using computational fluid dynamics (CFD) is proposed and developed. The PBA was based on an extension of Murray’s law and additional inlet conditions prescribed iteratively and was implemented in OpenFOAM v1912 for testing and validation. 3D models of CATs were created using CT scans and computational meshes, and the results were compared to invasive coronary angiographic (ICA) data to validate the accuracy and effectiveness of the PBA. The discrepancy between the calculated and experimental FFR was within 2.33–5.26% in the steady-state and transient simulations, respectively, when convergence was reached. The PBA was a reliable and physiologically sound technique compared to a current lumped parameter model (LPM), which is based on empirical scaling correlations and requires nonlinear iterative computing for convergence. The accuracy of the PBA method was further confirmed using an FDA nozzle, which demonstrated good alignment with the CFD-validated values.

## 1. Introduction

The coronary artery is one of the essential blood arteries that ensure the heart receives steady blood flow. The heart muscle (myocardium) receives oxygenated blood via the coronary arteries; when these are clogged or obstructed, the myocardium starts to deteriorate, a condition known as ischemia. The most significant cause of death worldwide is coronary artery disease (CAD), also known as ischemic heart disease. According to a report by the World Health Organization, CAD causes nearly 50% of disease-related deaths in Kazakhstan [1]. An American experience a coronary episode roughly every 25 s; statistically, the pass-away rate is approximately one individual every minute [2].

Coronary angiography may offer information on anatomical stenosis, the gold standard for diagnosing coronary heart disease. Evaluations of fractional flow reserve (FFR) from CT in Europe and the United States suggest that it has the potential to decrease false diagnoses before patients are referred for invasive testing [3].

Coronary artery computerized tomography (CT) imaging is preferred over coronary angiography for prescreening asymptomatic individuals due to its noninvasive nature, ease of use, low cost, and excellent repeatability. Since FFR from CT is based on high-quality coronary artery CT angiography (CTA) image data, it does not need extra loading, scanning, or dosage. Although there is a grey area that necessitates additional functional measures for diagnosis, the diagnostic accuracy and specificity are greater than those of coronary artery CT imaging. The quantitative coronary artery CT-imaging index may objectively identify functional coronary stenosis [4].

Compared to angiography alone, FFR is one of the few diagnostic tools that can effectively lead to therapeutic approaches, improve safety and efficacy, and reduce costs. Angio-FFR, a noninvasive estimate of FFR generated from computed tomography coronary angiography, employs specialized software to model three-dimensional coronary blood flow. The DISCOVER-FLOW research revealed an 84.3 percent diagnosis accuracy for lesions contextually assessed using FFR. Similarly, the HEARTFLOW-NXT research found an 86 percent per vessel diagnosis accuracy [5].

As an optional tool for hemodynamic evaluation in patients with single-vessel CAD, the excellent diagnostic accuracy of SPECT CFR for diagnosing functionally significant stenosis justifies its usage. SPECT CFR is supported by superior diagnostic accuracy for identifying functionally significant stenosis. An aberrant CFR may suggest microvascular dysfunction in individuals whose FFR and CFR differ, which needs more research [6].

CT scans may be used to construct three-dimensional (3D) solid models that can be seen on display, printed on film or by a 3D printer, or used by a computational fluid dynamics (CFD) approach that enables doctors to quantify the coronary physiology inside an artery. Current CFD programs that have undergone clinical examination in significant clinical investigations include Discover Flow, HeartFlow—NXT, and other platforms for the physiologic study of coronary artery function [7]. Additionally, using CFD to estimate FFR was offered as a potential noninvasive alternative to fractional flow reserve invasive coronary angiography measurement [8].

Consequently, computational analysis of FFR utilizing CCTA imaging data enables noninvasive, lesion-specific decline assessment. This anatomical and functional assessment may identify people with lesion-causing coronary pressure decreases. This noninvasive approach may be superior to invasive ICA with FFR for patient treatment. We need more information on incorporating this new strategy into “real-world” clinical practice to influence future patient care decisions [9].

Instead of simulating maximum hyperemia, boundary conditions are specified to create a pressure–flow curve for stenosis. Then, stenosis is functionally diagnosed using pressure–flow curve characteristics. The suggested strategy is verified with invasive FFR in six individuals using idealized and patient-specific models. According to the results, stenosis flow resistances cannot be directly acquired from anatomy. Simulated pressure–flow parameters correlate linearly and significantly with invasive FFR. The suggested technique may estimate flow resistances using pressure flow from curve-derived parameters. Furthermore, flow resistances can be assessed without modeling maximum hyperemia [10].

However, since a cardiac tree often includes multiple capillary branches connecting with downstream microcirculation, it is almost impossible to measure the outflow border conditions experimentally owing to their tiny branch diameters. Consequently, the Windkessel-type boundary conditions are based on the so-called lumped parameter model (LPM) and the more complex lumped parameter network model (LPNM). Both methods are typically adopted for approximating the outflow boundary conditions that represent the highly complex dynamic interactions between a tree and its downstream microvasculature. These approaches are based on the circuit analogy theory, which necessitates the measurement of resistances, capacitances, and empirical correlations, which are sometimes challenging to compute without patient-specific data and are not physiologically grounded. The connection of the generated ordinary differential equations (ODEs) from these approaches with the CFD solver also creates unclear boundary conditions, which may often result in slow convergence or even divergence of numerical solutions [11].

One of the recent works conducted by Chakshu et al [12] represented a new technique for decreasing invasive, catheter-based assessments consisting of a quick approach for calculating FFR from CT images without operator input that was powered by unsupervised learning and combined with computational fluid dynamics.

The problem addressed in this work is the diagnosis of stenosis (obstruction or narrowing) in the coronary arteries of individual patients. Traditional methods for diagnosing stenosis, such as coronary angiography, have limitations and can be invasive and costly and can carry risks for patients. There is a need for more effective and noninvasive diagnostic methods for stenosis.

The PBA method proposed in this work is based on an extension of Murray’s law and different inlet criteria [13] and is designed to be used alternately and iteratively to compute the outflow boundary conditions of coronary trees for individual patients. The PBA method is validated using FFR measurements in actual patient arteries and benchmarking with an FDA nozzle. It is intended to provide realistic and tailored outflow boundary conditions for the diagnosis of stenosis without requiring measurable data. The unique contribution of the PBA method to the field is its use of Murray’s law to establish initial boundary conditions that are then modified through the addition of new inlet boundary conditions and iterations, resulting in numerical convergence. This approach may provide a more effective and noninvasive method for diagnosing stenosis in the coronary arteries of individual patients.

In the cardiovascular system, agreements between experiments and the Murray diameter model have been found only in small arteries [14] and arterioles [15,16], as present in our CAD case. The final conditions at the outlets are ultimately decided by a tree’s geometry, the conservation laws built into CFD, the numerical iterations, and the additional inlet patient-specific parameters; we only utilize Murray’s formula to estimate their initial conditions. The third law may not accurately predict the outcome since we use it as a preliminary approximation.

Unlike machine-learning-based data-driven simulation models, which depend on a significant quantity of data for verification, this PBA model is entirely based on physiology and physics. We are currently in the conceptual model-proof stage. It is undoubtedly desirable to undertake large-scale clinical testing, which we hope to accomplish in the next step for possible commercialization and future clinical uses. Because of this, we perform model validation using experimental benchmark data and outputs from other simulations.

The IREC approved the study related to patient data collection and ethical approval.

## 2. Mathematical Formulations and Numerical Methods

### 2.1. Governing Equations for Hemodynamic Flow

OpenFOAM v1912 is software used to solve fluid flow problems by numerically solving the governing equations for blood flow using the SIMPLE and PIMPLE algorithms. These algorithms can be used to perform steady-state and transient simulations, respectively, and are capable of handling 3D laminar, turbulent, steady, and unsteady flows and compressible and incompressible flows. When calculating blood flow in OpenFOAM, the software uses the incompressible 3D Navier–Stokes formula (Equation (1)) to model the flow of the fluid.

(1) $$\frac{\partial u}{\partial x}+\frac{\partial v}{\partial x}+\frac{\partial w}{\partial x}=0\;\rho \left(\frac{\partial u}{\partial t}+u\frac{\partial u}{\partial x}+v\frac{\partial u}{\partial y}+w\frac{\partial u}{\partial z}\right)=-\frac{\partial P}{\partial x}+\rho g_{x}+\mu \left(\frac{\partial^{2}u}{\partial x^{2}}+\frac{\partial^{2}u}{\partial y^{2}}+\frac{\partial^{2}u}{\partial z^{2}}\right)\;\rho \left(\frac{\partial v}{\partial t}+u\frac{\partial v}{\partial x}+v\frac{\partial v}{\partial y}+w\frac{\partial v}{\partial z}\right)=-\frac{\partial P}{\partial y}+\rho g_{y}+\mu \left(\frac{\partial^{2}v}{\partial x^{2}}+\frac{\partial^{2}v}{\partial y^{2}}+\frac{\partial^{2}v}{\partial z^{2}}\right)\;\rho \left(\frac{\partial w}{\partial t}+u\frac{\partial w}{\partial x}+v\frac{\partial w}{\partial y}+w\frac{\partial w}{\partial z}\right)=-\frac{\partial P}{\partial z}+\rho g_{z}+\mu \left(\frac{\partial^{2}w}{\partial x^{2}}+\frac{\partial^{2}w}{\partial y^{2}}+\frac{\partial^{2}w}{\partial z^{2}}\right)\;\rho \frac{\partial V}{\partial t}=-\nabla P+\rho g+\mu \nabla^{2}V\;\;\;\;\;\;\;\;\;\rho V_{i,t}+\rho \upsilon_{j}\upsilon_{i,j}-p_{,i}-\tau_{ij,j}=0,$$

(2) $$\upsilon_{i,i}=0$$

ρ—density of blood, kg/m^3^;

$\upsilon_{i}$—i component of velocity, m^3^/s;

$V_{i,t}$—velocity derivative with respect to time;

p—pressure, Pa;

$\tau_{ij}$—stress tensor (viscous portion).

OpenFOAM uses the finite volume method (FVM) and SIMPLE/SIMPLEC/PISO pressure correction schemes to solve the Navier–Stokes equations for incompressible fluid flow. The domain of the equation is defined as Ω in three dimensions, and the boundary conditions are specified as $\Gamma =\Gamma_{D}\cup \Gamma_{N}$, where $\Gamma_{D}$ represents Dirichlet boundary conditions and $\Gamma_{N}$ represents Neumann boundary conditions. The domain is discretized using $n_{el}$ linear, unstructured tetrahedral elements,${\overset{-}{\Omega }}_{e}$. The final form of the equation is given by Equation (3).

(3) $$B_{G}\left(w_{i},q;v_{i},p\right)=\int_{\Omega }\{w_{i}\left(\rho {\dot{v}}_{i}+\rho v_{j}v_{i,j}\right)+w_{i,j}\left(-\rho \sigma_{ij}+\tau_{ij}\right)-q_{,i}v_{i}\}d\Omega \;+\int_{\Gamma_{N}}\{w_{i}\left(\rho \delta_{in}+\tau_{in}\right)+qv_{in}\}d\Gamma$$

where $w\in W_{n}^{k}\;and\;q\in P_{h}^{k}$.

### 2.2. Murray’s Law and PBA for Rapid Iterative Computation of Outlet Conditions

The present work proposes a method that aims to address the problems related to the Windkessel-type boundary conditions and to provide patient-specific and realistic outflow boundary conditions in the absence of observed data. This method is based on an extension of Murray’s law and the use of different inlet conditions that are alternately and iteratively applied. Sumbekova et al. previously carried out experiments using commercial software [17]. It has been shown that vascular systems follow Murray’s law, which states that mammalian vascular transport systems use minimum energy for blood maintenance and transport.

According to the Hagen–Poiseuille Law for laminar flow in a vessel, the power required to drive blood flow through a vessel is as follows.

(4) $$P_{t}=\frac{8\mu l}{\pi r^{4}}{\dot{Q}}^{2}$$

where $\dot{Q}$ is the volumetric flow rate, *l* is the length of a vessel, *r* is its radius, and μ is blood viscosity.

Furthermore, the power necessary for the maintenance of the blood in a vessel is proportional to the blood volume in the vessel.

(5) $$V=\pi lr^{2}$$

The power required to maintain the metabolism in the blood is, thus, calculated as follows.

(6) $$P_{m}=\lambda V=\lambda \pi lr^{2}$$

where λ is the metabolic rate of blood.

The total power required to drive the blood and maintain it is calculated as follows.

(7) $$P=P_{t}+P_{m}=\frac{8\mu l}{\pi r^{4}}{\dot{Q}}^{2}+\lambda \pi lr^{2}$$

The radius that meets the minimum power requirement is obtained through the differentiation of *P* with respect to *r* set to zero.

(8) $$\frac{dP}{dr}=-\frac{32\mu l}{\pi r^{5}}{\dot{Q}}^{2}+2\lambda \pi lr=0$$

Thus:

(9) $$\dot{Q}=\frac{\pi }{4}r^{3}\sqrt{\frac{\lambda }{\mu }}$$

**Figure 1:**
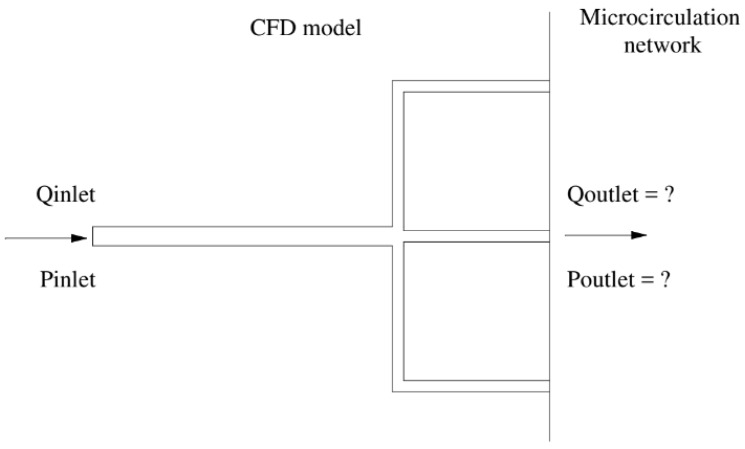
Schematic of the new boundary model.

The vessel radius and volumetric flow rate in individual vessels have this functional connection. To find the converged patient-specific flow outlet conditions in all the outlets of a coronary tree for a given inlet pressure and volumetric flow rate that are prescribed alternatively through iterative computation, a novel iterative scheme is, thus, proposed to couple Murray’s law with CFD simulation. For a particular branch *i* of a coronary tree with *N* branches, as shown in Figure 1, its outflow is as follows.

(10) $${\dot{Q}}_{i}=\frac{\pi }{4}r_{i}^{3}\sqrt{\frac{\lambda }{\mu }}$$

According to the conservation law, we have the following.

(11) $${\dot{Q}}_{in}=\sum_{i=1}^{N}{\dot{Q}}_{i}=\frac{\pi }{4}\sqrt{\frac{\lambda }{\mu }}\sum_{i=1}^{N}r_{i}^{3}$$

Equation (11) can then be obtained by dividing Equation (10) by Equation (11) and rearranging it:

(12) $${\dot{Q}}_{i}=\frac{r_{i}^{3}}{\sum_{i=1}^{N}r_{i}^{3}}{\dot{Q}}_{in}$$

**Figure 2:**
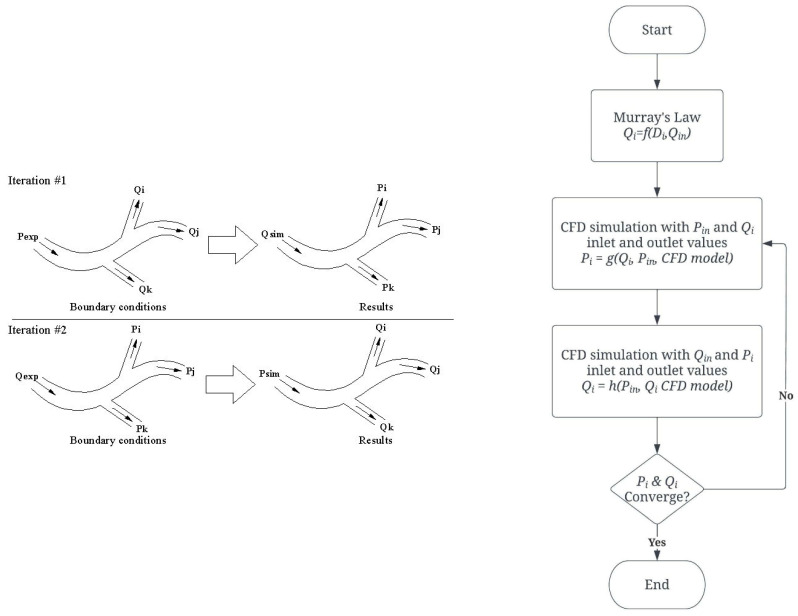
Algorithm for the outflow boundary conditions.

Our new physiologically based algorithm (PBA) aims to extract personalized, patient-specific outflow boundary conditions. It represents the interactions between a coronary tree and its microcirculation network downstream under both steady and unsteady conditions, as shown in a flowchart and illustrated in Figure 2.

In this work, the above-mentioned methods were implemented in OpenFOAM to perform a computational study of the hemodynamics in several patient-specific geometries with the aim of validating the methods using related ICA measurements, such as inlet flow rate, pressure, and FFR_ICA_. There were four vascular cases, named CT209, CHN13, CHN03, and FDA nozzle, implemented using the proposed PBA technique. Table 1 lists the workflow procedure for the PBA.

**Figure 3:**
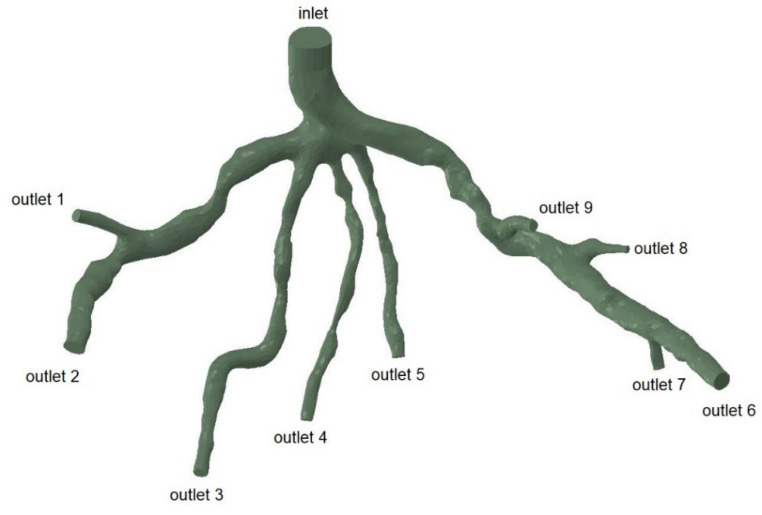
Geometry of inlet and outlets of the CT209 model.

**Figure 4:**
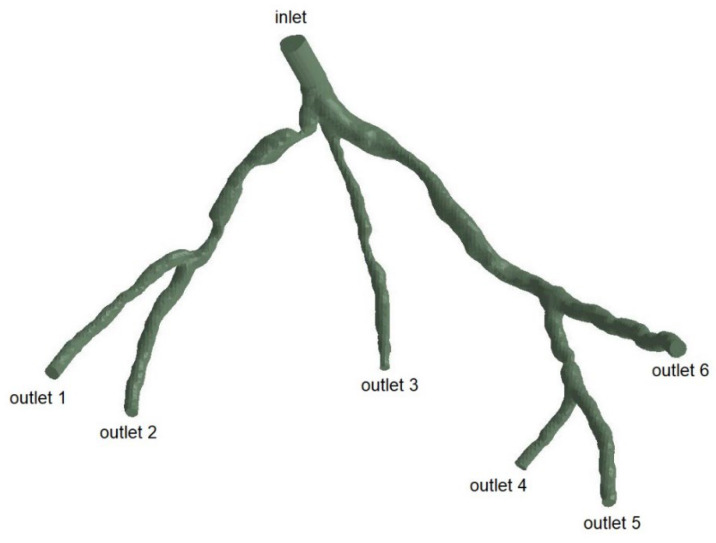
Geometry of inlet and outlets of the CHN13 model.

**Figure 5:**
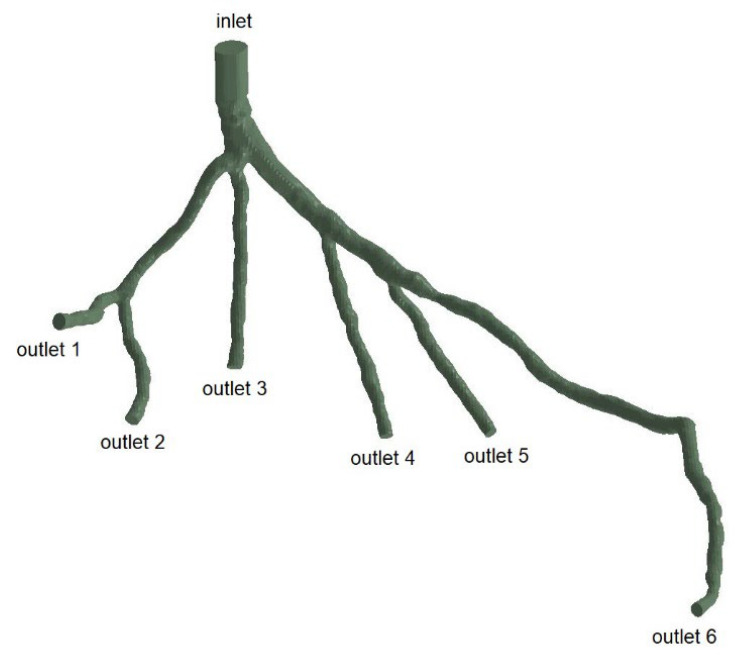
Geometry of inlet and outlets of the CHN03 model.

The geometries and outlets of CT209, CHN13, and CHN03 are presented in Figure 3, Figure 4 and Figure 5, respectively.

**Figure 6:**
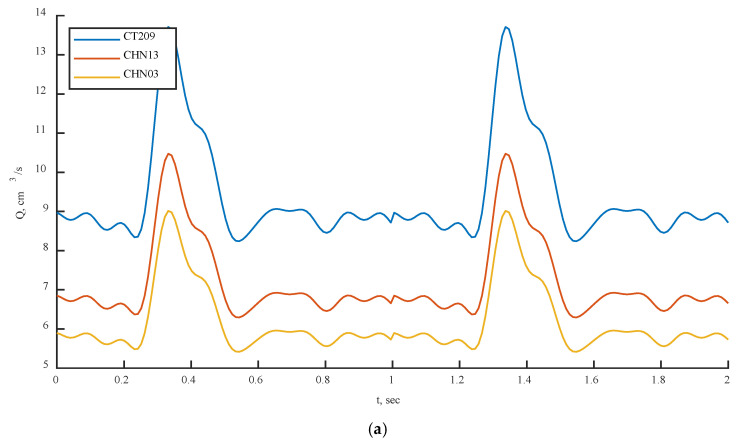

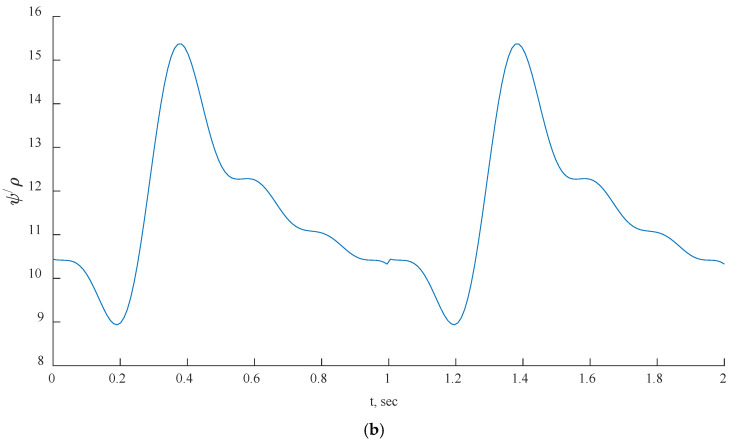
Inlet boundary conditions: transient flow rate (**a**) and normalized pressure (**b**) waveform of coronary blood flow.

Table 2, Table 3 and Table 4 present the experimental results of pressure and flow rate at the inlet of a vessel (aorta) for the steady-state simulations of the three artery models: CT209, CHN13, and CHN03. Similarly, Table 5, Table 6 and Table 7 display the initial flow rates at the outlets of the three artery models, which were calculated using Equation (11) based on Murray’s law with the inlet and outlet boundary conditions specified in Table 1. Figure 6 presents the inlet values for the transient case. The time-averaged value of the inlet waveform for the transient scenario was compared to the steady-state case to ensure the accuracy of the comparison.

In this study, the same set of fluid properties and blood models were used for both steady-state and transient simulations. The properties included a Newtonian dynamic viscosity of 0.0035 Ns/m^2^ and a density of 1056 kg/m^3^ [18]. The boundary conditions for these simulations included velocity/pressure inlets and velocity/pressure outlets, which were iteratively switched as part of the PBA scheme. The blood flow was modeled as laminar fluid flow. To ensure convergence, the criteria for the transient simulations required that the residual reduction must be lower than five orders of magnitude, ranging from 1 down to 10^−5^, while the criteria for the steady-state simulations required that the residual reduction must be lower than 10^−3^. In addition, the inlet data provided for each artery indicated that two cycles were used in the transient case.

**Table 2:** Experimentally obtained input parameters for simulation of CT209.

Parameter	Value
Experimental inlet pressure $P_{exp}$	90.53 mm Hg (12,070.12 Pa)
Experimental inlet flow rate $Q_{exp}$	9.39944 cm^3^/s

**Table 3:** Experimentally obtained input parameters for simulation of CHN13.

Parameter	Value
Experimental inlet pressure $P_{exp}$	90.61 mm Hg (12,870.12 Pa)
Experimental inlet flow rate $Q_{exp}$	7.17551 cm^3^/s

**Table 4:** Experimentally obtained input parameters for simulation of CHN03.

Parameter	Value
Experimental inlet pressure $P_{exp}$	76.5 mm Hg (10,201.9 Pa)
Experimental inlet flow rate $Q_{exp}$	6.18 cm^3^/s

**Table 5:** Initial calculated flow rates at each outlet by Murray’s law for CT209 [19].

Murray’s Law Calculation for Outlet Flow Rates					
	$A_{i}$ (cm^2^)	$d_{i}$ (cm)	$d_{i}^{3}$ (cm^3^)	$\alpha_{i}$	$Q_{i}$ (cm^3^/s)
outlet 1	1.674	1.46	3.111	0.082	0.769
outlet 2	4.105	2.286	11.948	0.314	2.955
outlet 3	1.802	1.515	3.475	0.091	0.859
outlet 4	0.977	1.116	1.388	0.037	0.343
outlet 5	1.398	1.334	2.374	0.062	0.587
outlet 6	4.114	2.289	11.988	0.315	2.965
outlet 7	0.925	1.085	1.279	0.034	0.316
outlet 8	0.568	0.851	0.616	0.016	0.152
outlet 9	1.173	1.222	1.824	0.048	0.451

**Table 6:** Initial calculated flow rates at each outlet by Murray’s law for CHN13 [19].

Murray’s Law Calculation for Outlet Flow Rates					
	$A_{i}$ (cm^2^)	$d_{i}$ (cm)	$d_{i}^{3}$ (cm^3^)	$\alpha_{i}$	$Q_{i}$ (cm^3^/s)
outlet 1	2.712	1.858	6.418	0.227	1.630
outlet 2	1.832	1.527	3.564	0.126	0.905
outlet 3	1.005	1.131	1.447	0.051	0.368
outlet 4	1.950	1.576	3.913	0.139	0.994
outlet 5	1.969	1.583	3.970	0.141	1.009
outlet 6	3.382	2.075	8.936	0.316	2.270

**Table 7:** Initial calculated flow rates at each outlet by Murray’s law for CHN03 [19].

Murray’s Law Calculation for Outlet Flow Rates						
	i	$A_{i}$ (cm^2^)	$d_{i}$ (cm)	$d_{i}^{3}$ (cm^3^)	$\alpha_{i}$	$Q_{i}$ (cm^3^/s)
outlet 1	1	2.5675	1.8081	5.9106	0.2240	1.3847
outlet 2	2	2.2262	1.6836	4.7721	0.1808	1.1179
outlet 3	3	2.1930	1.6710	4.6658	0.1768	1.0930
outlet 4	4	1.8206	1.5225	3.5293	0.1338	0.8268
outlet 5	5	1.7784	1.5048	3.4074	0.1291	0.7982
outlet 6	6	2.0126	1.6008	4.1021	0.1555	0.9610

**Figure 7:**
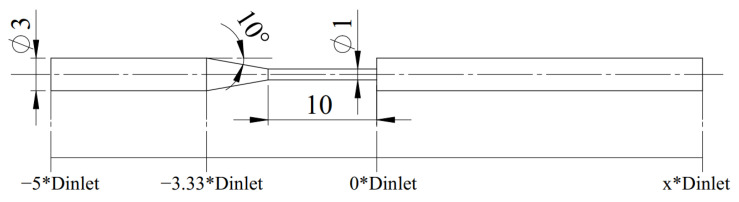
Geometry of coronary nozzle [18].

**Figure 8:**
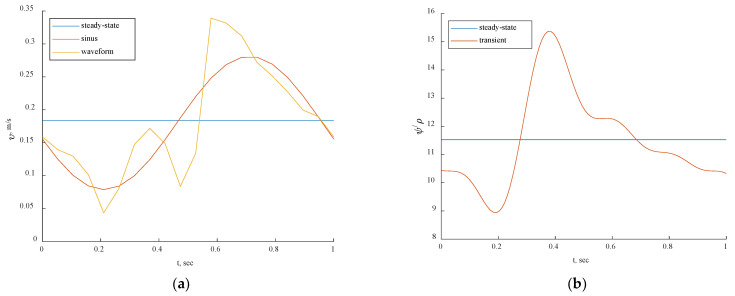
(**a**) Inlet boundary conditions: steady-state velocity waveform of left main coronary blood flow (adapted from [22]) and sinus flow: *u(t)* = 0.184 m/s − 0.02432 m/s*cos(2π*t) − 0.09822m/s*sin(2π*t). (**b**) Inlet boundary conditions: normalized steady-state pressure and normalized transient waveform pressure.

The PBA study was replicated on an FDA benchmark model to validate the PBA approach. The nozzle geometry used in this study is shown in Figure 7 and has been previously described in detail by Stewart et al. [20,21]. The original FDA nozzle had a 12 mm diameter inlet and outlet tube, with a throat-to-inlet tube ratio of 1:3. Stiehm et al. scaled down the nozzle geometry to a 3 mm tube diameter. Normalization of the results was performed to ensure comparability with the outcomes of the FDA’s round-robin trial. The FDA nozzle in this study only used one cycle, as the same boundary conditions were already tested by Stiehm et al. [18]. The input parameters for simulating an FDA nozzle using the PBA approach for steady-state and transient cases can be found in Table 8 and Figure 8, respectively.

The convergence criteria were evaluated at the end of each iteration with Equation (13). The procedure presented in Table 1 was repeated continuously until convergence was achieved. FFR was computed using Formula (13).

(13) $$FFR=\frac{\min\left(P_{1}^{},P_{2}^{},\ldots P_{n}^{}\right)}{P_{exp}}$$

## 3. Results and Discussion

**Figure 9:**
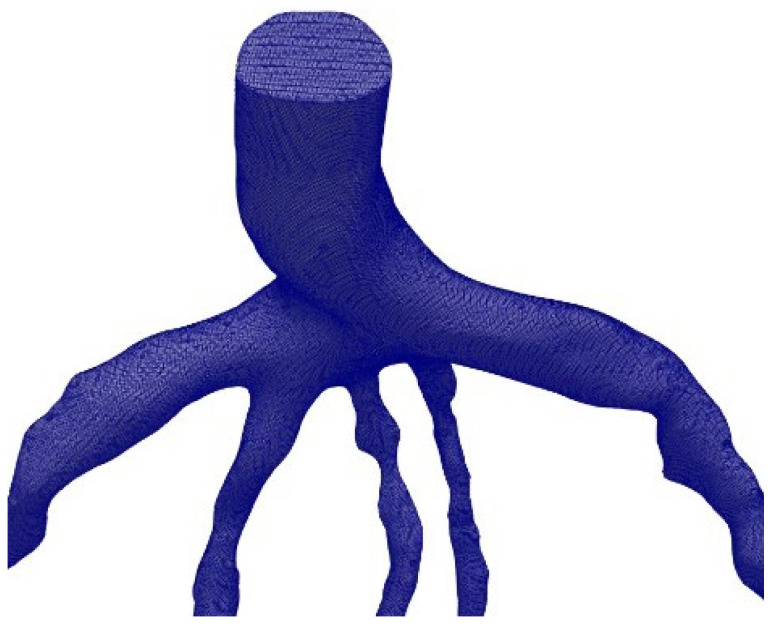
Cartesian mesh example.

The mesh generation for the blood vessels was performed using the open-source cfMesh utility, which is part of OpenFOAM and generates volumetric meshes of unstructured Cartesian types. cfMesh is also able to automatically generate tetrahedral meshes for a variety of geometries, as shown in Figure 9. A mesh sensitivity analysis was conducted [19], and the same models were used in this study. For the CT209, CHN13, and CHN03 arteries, our research group [19] calculated mesh sizes and numbers of grid cells based on the results of the mesh sensitivity analysis.

The minimum mesh size was half the radius of the model’s smallest artery (inlet or outlet). CT209’s mesh size was 0.1702 mm, whereas CHN13’s was 0.2262 mm. Mesh refinement resulted in a larger number of mesh elements without expansion layers along the vessel walls, which increased simulation computational time. Decreased mesh size from 0.20 mm to 0.1702 mm resulted in 1.7 times increase in computational time.

CHN03 had five meshes (69,681; 139,833; 286,897; 406,606; and 779,482) to examine mesh convergence. Increasing the grid number from 100,000 to 1.05 million decreased distal pressure by 2.2%. The model was discretized with 0.5 million volume cells after mesh dependency testing. At 0.4 million volume cells, the CHN03 mesh converged. In Table 9 and Table 10, respectively, mesh details for the CT209 and CHN13 artery models are shown.

**Figure 10:**
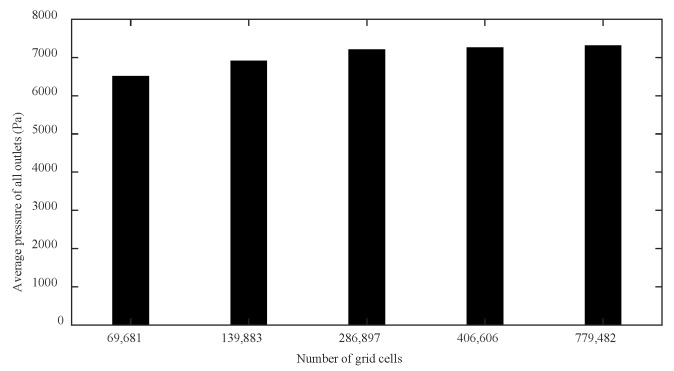
Dependency analysis for CHN03 [19].

There were five sets of meshes for CHN03 (with mesh element numbers of 69,681; 139,833; 286,897; 406,606; and 779,482) to study mesh convergence. Figure 10 shows that, when the grid number was increased from 100,000 to about 1.05 million, the change value of the distal pressure was less than 5% (about 2.2%). After mesh dependency test, the model was discretized with a total of about 0.5 million volume cells. Further grid refinement led to <1% relative error. For CHN03, the mesh convergence results are shown in Figure 10. The number of volume cells was finally set to 0.4 million.

**Figure 11:**
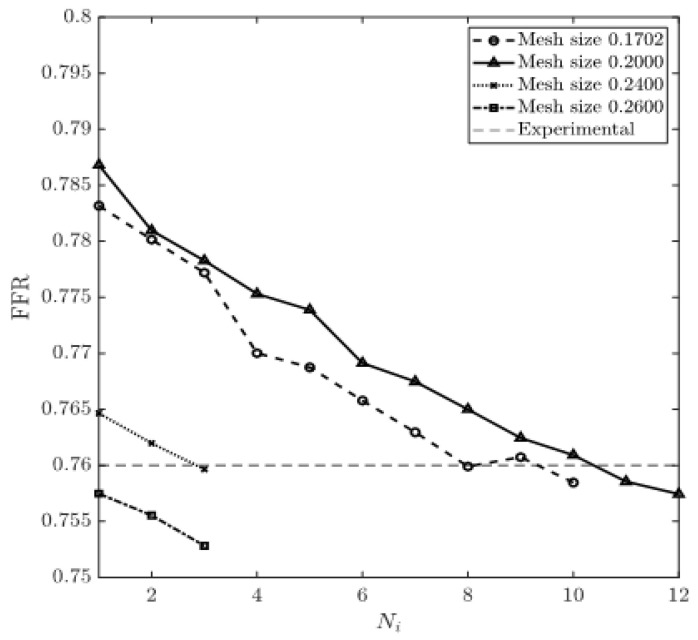
FFR values in each round of iteration for CT209 [19].

**Figure 12:**
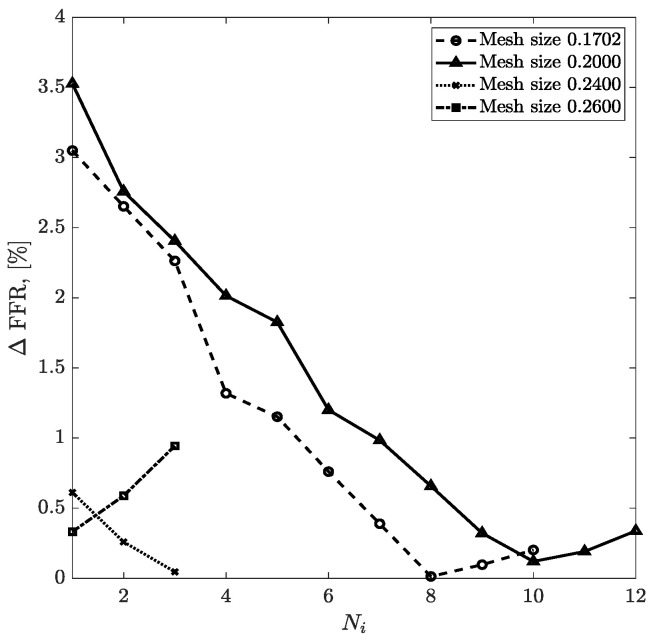
Relative differences between calculated and experimental FFR in each round of iterations for CT209 [19].

Figure 11 and Figure 12 show the obtained values of FFR at each round of iteration and the percentage relative difference of the calculated and experimental FFR (degree of deviation from experimentally obtained FFR). The results obtained for mesh sizes of 0.1702 and 0.20 were very close to the experimental value of FFR = 0.76 at the end of the eighth and the tenth rounds of iteration, respectively. At the same time, the results obtained for mesh sizes from 0.24 to 0.26 mesh demonstrated a constant decrease, as shown in Figure 11, which yielded a constant negative slope. FFR values for mesh sizes between 0.1702 and 0.20 converged when they reached the experimental FFR value. The percentage relative differences of 0.20% and 0.34% for FFR values were recorded for the mesh sizes of 0.1702 and 0.20, respectively.

**Figure 13:**
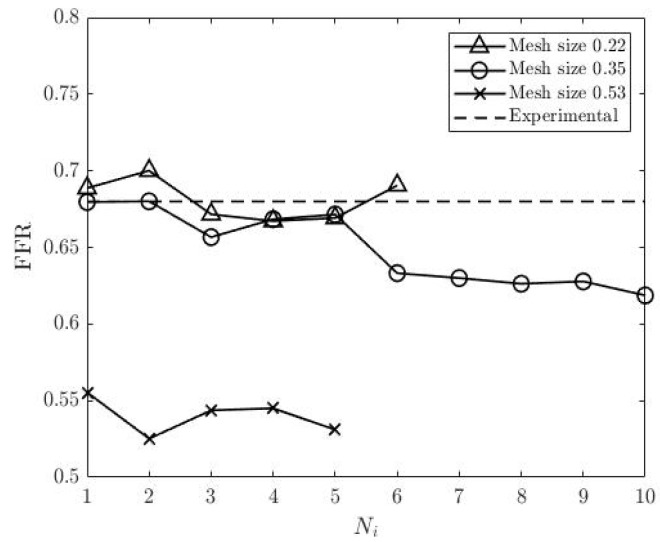
FFR values in each round of iteration for CHN13 [19].

**Figure 14:**
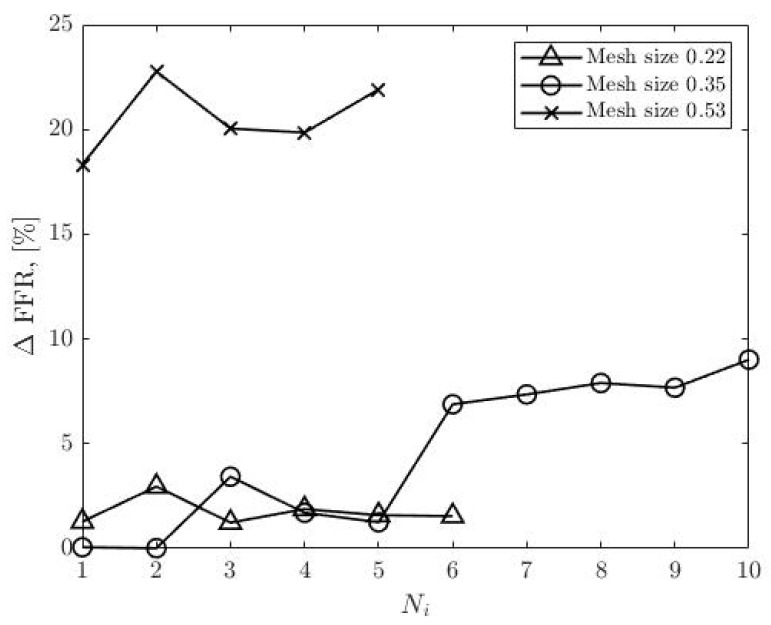
Relative differences between calculated and experimental FFR in each round of iteration for CHN13 [19].

The simulations of blood flow in the CHN13 model were performed using three mesh sizes (coarse, fine, and very fine). Initially, the number of iterations was set to ten; however, since the relative errors for some mesh sizes showed systematic increases, the simulations with 0.53 and 0.22 mesh sizes were stopped at the fifth and sixth rounds of iterations, respectively. The experimental FFR for the CHN13 model was 0.68. The results showed that the finest mesh size of 0.22 mm produced the closest amount to this value. The FFR obtained from the simulations with the finest mesh size was equal to 0.691, as shown in Figure 13. The final values of FFR obtained using the 0.35 and 0.53 mesh sizes were 0.619 and 0.531, respectively. Additionally, the results in Figure 14 show that the accuracy of the FFR obtained from simulations with the 0.35 mesh size decreased with the number of iterations. Consequently, the maximum error was observed in the tenth round of iterations.

**Figure 15:**
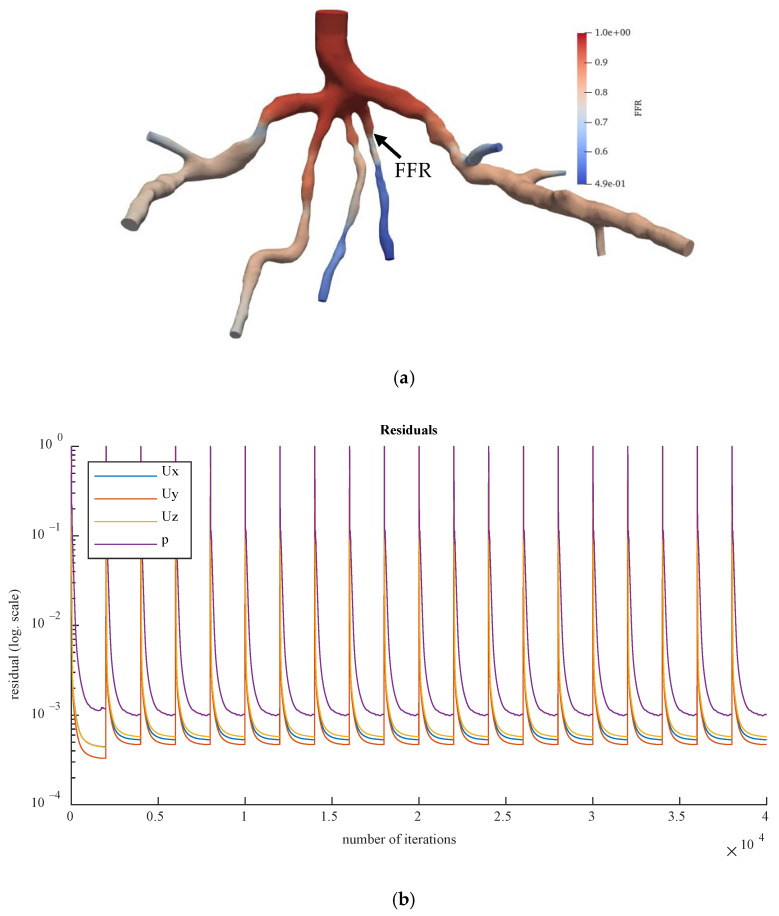
(**a**) FFR distribution with shape of CT209 steady-state PBA model and (**b**) PBA residual history.

The pressure and flow rate residuals at all the outlets were defined as the averages of relative percentage differences in consecutive iterations. The convergence of the CFD simulation could be monitored by examining the reduction in the values of pressure and velocity/momentum equation residuals at the outlets as well. Figure 15 reveals the pressure and flow velocity residuals at the outlets of CT209 for all the iterations, where residual values are plotted in logarithmic scale.

FFR was calculated using Equation (13), and for the CT209 model, it had an experimental value of 0.76 [23]. Figure 15 shows the derived FFR values from the most recent iteration, along with the relative percentage difference between the computed and experimental FFR (the degree of deviation from the experimentally obtained FFR). The calculated noninvasive FFR value of 0.73 by Zhang et al. [23] is different from the experimental (ICA) FFR value of approximately 0.76 for the CT209 model. After conducting a mesh sensitivity analysis, Sumbekova et al. [19] from our research group obtained a final FFR value of FFR = 0.757, which was very close to the ICA FFR. The same algorithm implemented in OpenFOAM produced results that were similar to the ICA measurement, with a final FFR value of FFR = 0.762.

According to Zhang et al. [23], the left anterior descending (LAD) proximal artery was the site of arterial stenosis in the CT209 model [23]. In Figure 15, the LAD proximal region is the dark blue area of the blood artery, and it was observed that the artery stenosis was in the same location as that identified by Zhang et al. [23]. Figure 15a includes the visualized FFR findings from the tenth iteration of the steady-state PBA.

**Figure 16:**
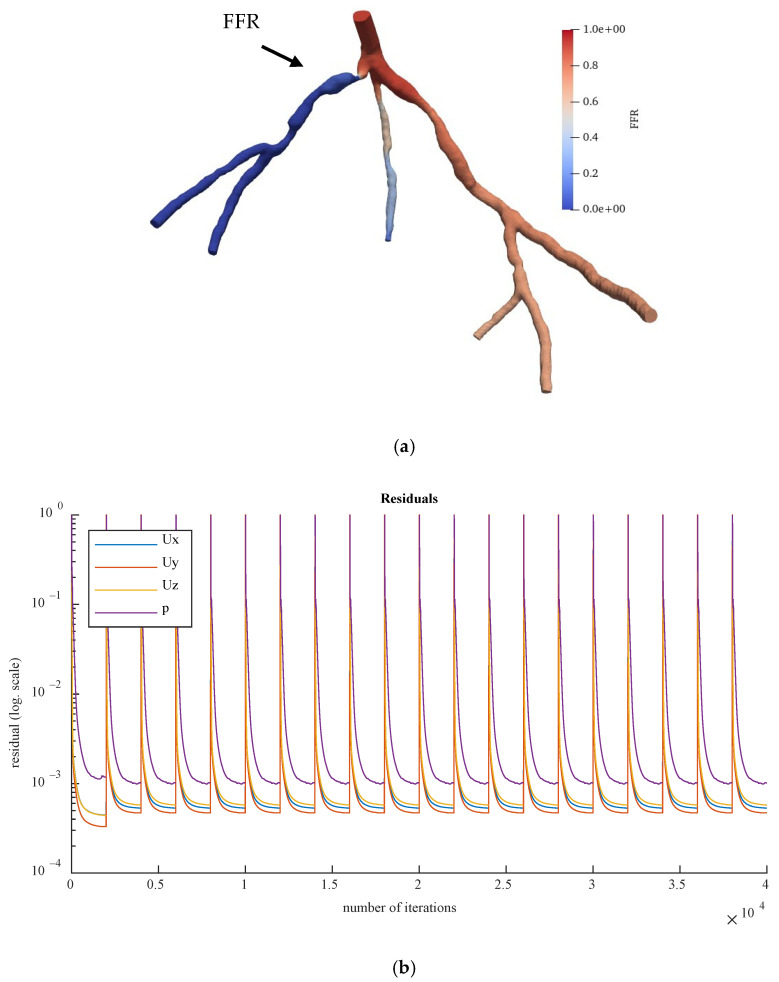
(**a**) FFR distribution with shape of CHN13 steady-state PBA model and (**b**) PBA residual history.

**Figure 17:**
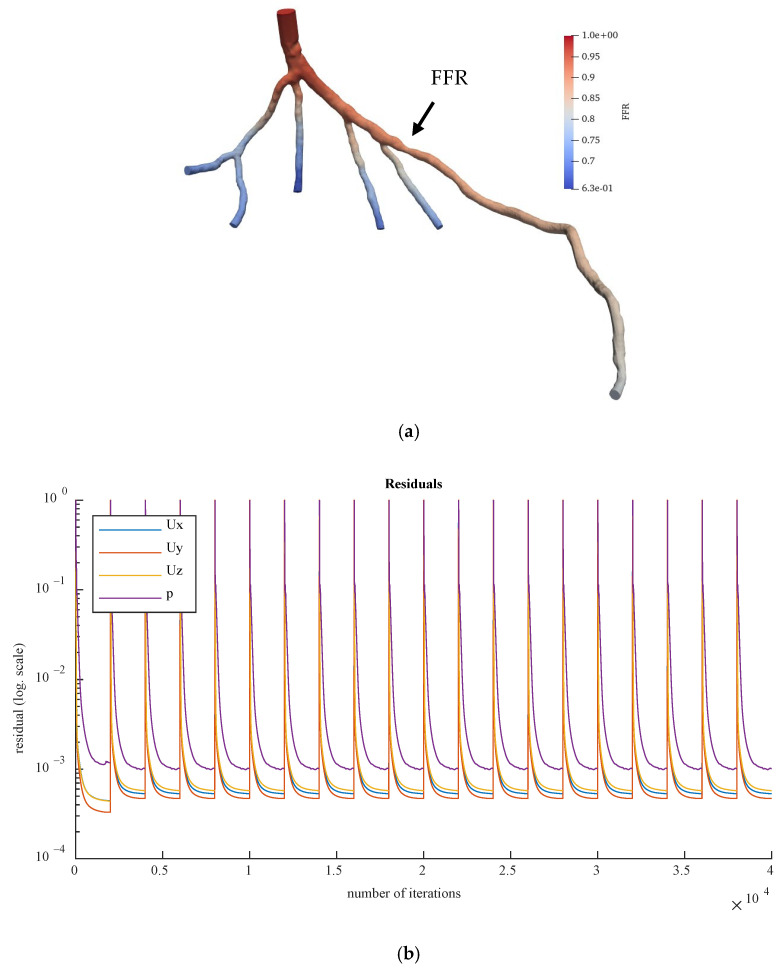
(**a**) FFR distribution with shape of CHN03 steady-state PBA model and (**b**) PBA residual history.

We compared the steady-state results obtained by the PBA method in OpenFOAM with those obtained using a traditional lumped parameter method and the current approach used by our research group [19], as well as with the ICA measurement results. It was found that the PBA method demonstrated good accuracy and efficiency, similar to the traditional methods. Figure 16 and Figure 17 show the visualized FFR distributions for the CHN13 and CHN03 models, respectively. The calculated FFRs for CHN03 and CHN13 by the OpenFOAM PBA were 0.88 and 0.683, respectively, which are in excellent agreement with the corresponding experimental values of 0.86 and 0.68.

**Figure 18:**
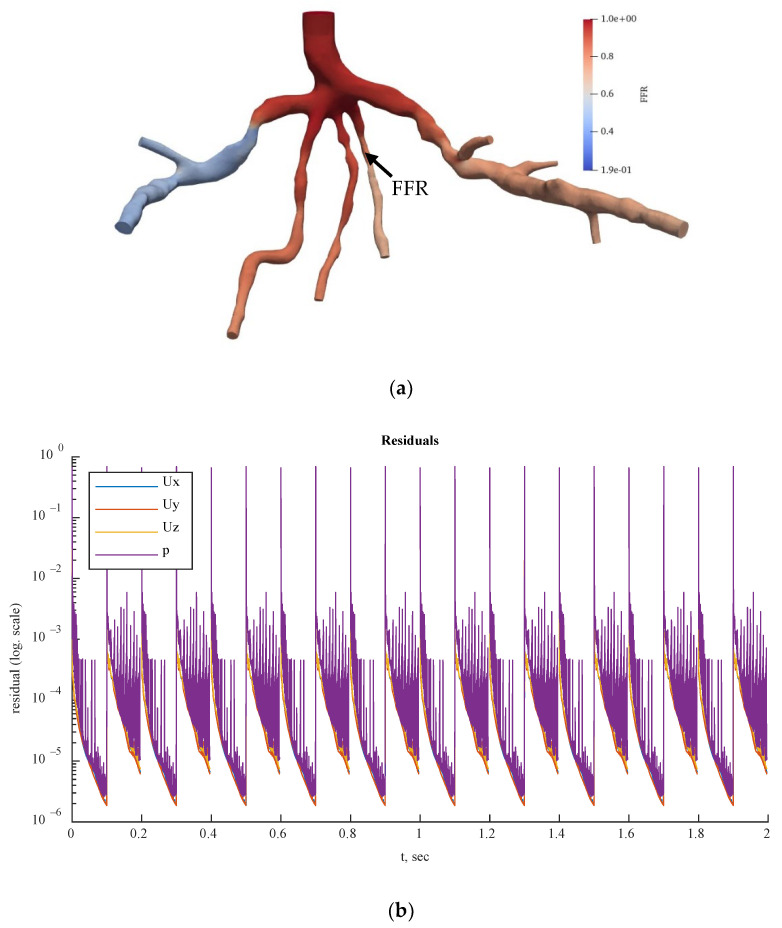
(**a**) FFR distribution with shape of CT209 transient PBA model and (**b**) PBA residual history.

**Figure 19:**
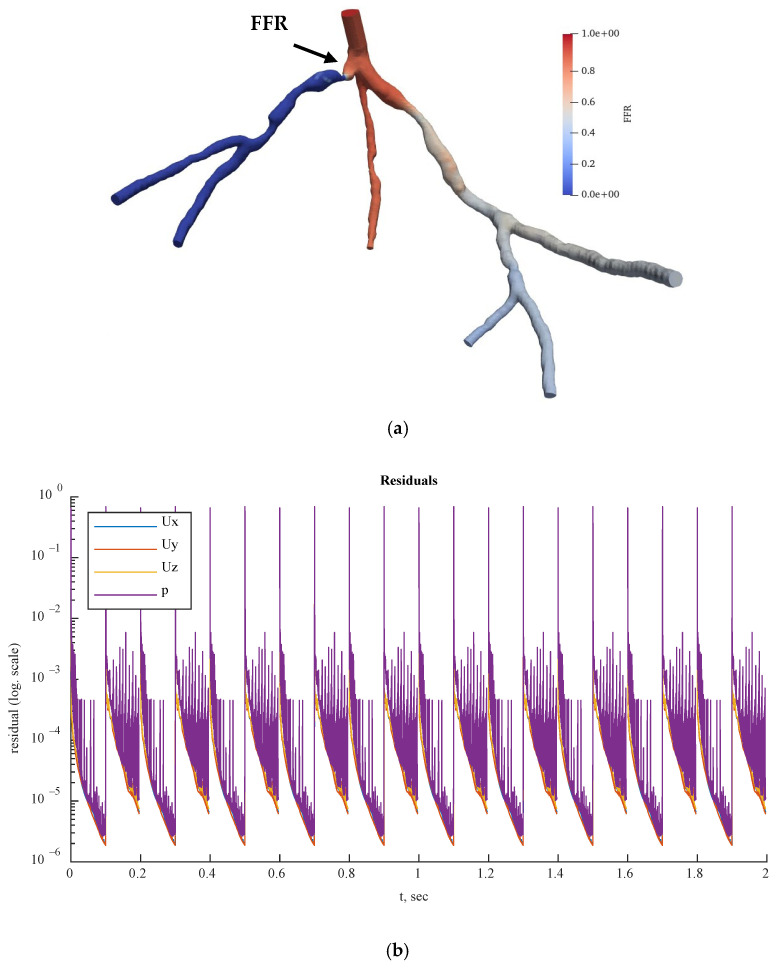
(**a**) FFR distribution with shape of CHN13 transient PBA model and (**b**) PBA residual history.

**Figure 20:**
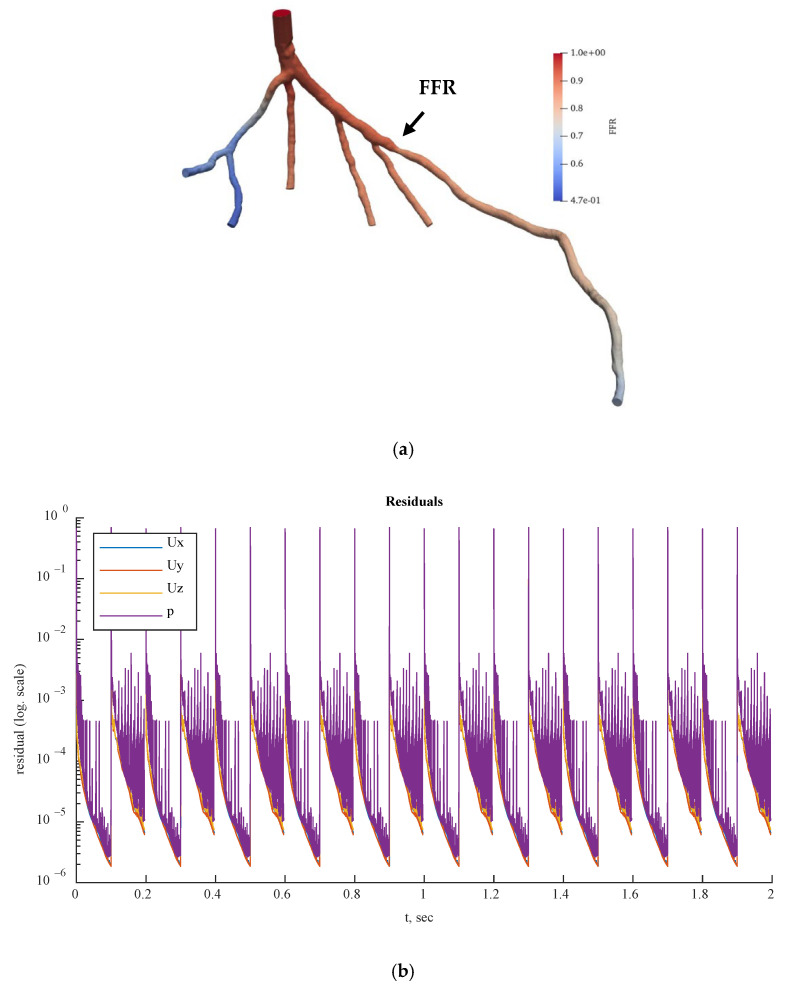
(**a**) FFR distribution with shape of CHN03 transient PBA model and (**b**) PBA residual history.

Figure 18, Figure 19 and Figure 20 show the FFR distributions in transient simulations for the CT209, CHN13, and CHN03 models, respectively. The PBA residuals also demonstrated convergence in the velocity and pressure equations.

Preliminary steady-state simulations were conducted to achieve convergence of the model before performing OpenFOAM transient PBA simulations using the mapFields package. The mapFields utility converts one or more fields that are specific to a geometry into their equivalents, and it is universal because there is no requirement for the geometries to be similar [17].

In Figure 18b, Figure 19b and Figure 20b, during iterations, the PBA method switched the boundary conditions, which could lead to high-frequency oscillations. For transient simulations, it was necessary to drive the solution to convergence at each timestep, which resulted in fluctuations that appeared to have high frequencies. The solution could exhibit wave reflections resembling high-frequency oscillations due to the complex geometry. Figure 16 compares the FFR values between steady-state and transient simulations at the locations indicated by the FFR arrows in Figure 16, Figure 17, Figure 18, Figure 19 and Figure 20. The FFR values increased in all three scenarios and tended to decrease due to alignment with the flow rate waveform.

**Figure 21:**
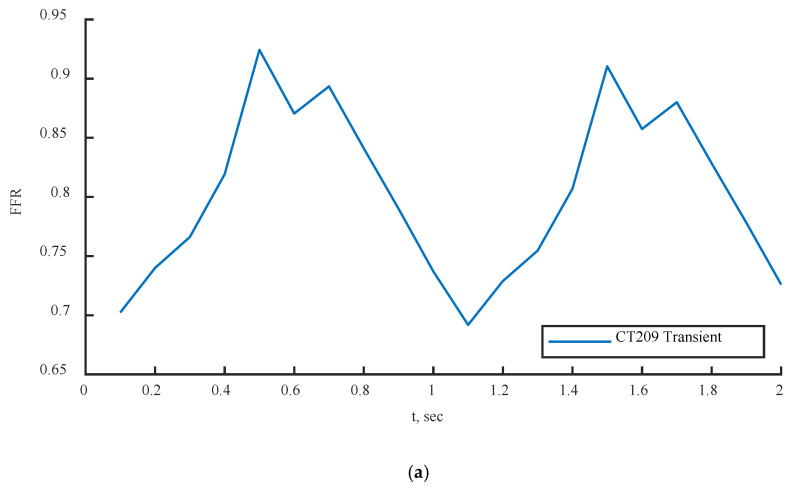

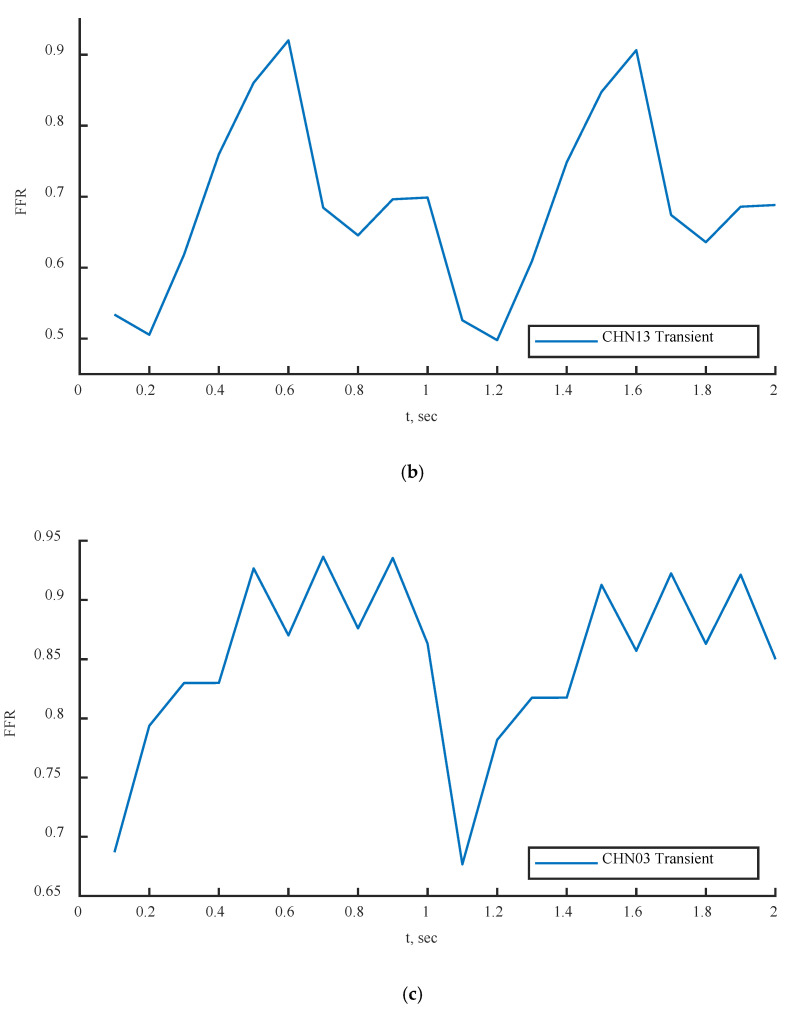
(**a**) FFR probe value comparison between steady-state and transient PBA models for (**a**) CT209, (**b**) CHN13, and (**c**) CHN03.

The mean FFR values are included in Table 11 as the outcome values for the transient simulations. As shown in Figure 21c, the second half of the transient instance exhibited rapid fluctuations in FFR due to changes in the PBA boundary conditions. In Figure 21a,b, the models show good agreement with the flow rate inputs, indicating that the PBA method could converge over several flow rate cycles.

Three artery models were analyzed using both methods: the standard LPM technique was used in ANSYS [23], while the suggested PBA was implemented in Simvascular [19] and OpenFOAM for steady-state and transient cases, respectively, as described in Appendix A and Appendix B. Additionally, all abbreviations in this work are provided in Appendix C.

Table 9 provides a summary of the comparison between the computed and experimental ICA FFRs [19]. This table shows that the recommended PBA produced results that were independent of the solver. Simvascular and OpenFOAM were used for the suggested PBA, while ANSYS was used for the conventional LPM approach.

The findings indicate that the relative errors or discrepancies across steady-state simulations in ANSYS CFX, Simvascular, and OpenFOAM did not exceed 3.24%. The results also show that the relative error disparities in transient simulations in OpenFOAM did not exceed 5.26%. The smallest inaccuracy was observed between the Simvascular and OpenFOAM steady-state PBA for CT209 at 0.26%. The largest inaccuracy for CT209 was in the OpenFOAM transient PBA at 5.26%. This may be due to the highest flow rate values, which could increase the overall FFR readings in the tested artery. The error in the OpenFOAM transient PBA for CHN03 was the smallest recorded at 0.02%, while the largest inaccuracy for CHN03 was in the OpenFOAM steady-state PBA at 2.33%. The error in the OpenFOAM steady-state PBA for CHN13 was the lowest at 0.44%, while the highest error was in ANSYS CFX at 3.24%. Despite the error peaks associated with various methods, the PBA approach showed a promising performance.

The classic LPM and LPNM techniques, which involve the calculation of capacitance, resistance, and inductance and the creation of a fictitious downstream capillary vessel network using fractal techniques, are fundamentally different from the PBA approach. The LPM and LPNM techniques require the calculation of resistance at the outlets and the use of additional inlet measurement conditions and numerical iterations, which are not based purely on physiology. In contrast, the PBA technique is patient-specific and physiologically based. It estimates the initial conditions at the outlets using Murray’s law and then uses the geometry of the arterial tree, the conservation laws incorporated into CFD, the suggested numerical iteration scheme, and the additional measured inlet patient-specific conditions to determine the final conditions at the outlets. The suggested PBA strategy, which is completely based on physics and physiology and is patient-specific, was also shown to be computationally effective. It was integrated into the standard CFD pressure correction iterations as an iterative boundary-switching system that did not require two simulation rounds.

The PBA approach was further validated using the standard LPM method published by Zhang et al. [23] for real-patient arteries. The LPM method employs a reference pressure, resistances at every outlet representing the flow resistance from the downstream microvasculature, and an overall resistance for the entire CAT that is related to outlet resistances through a population-averaged empirical scaling law [23]. The LPM is an iterative procedure that calculates the resistances and reference pressure to determine the outlet pressures in each branch outlet. It has been found that the LPM does not always ensure convergence to a unique solution for every situation. In contrast, the suggested PBA also achieved convergence to precise answers for all three analyzed scenarios. The PBA approach was found to be more reliable than the LPM because it is physiologically based and patient-specific, while the LPM does not consider these characteristics.

**Figure 22:**
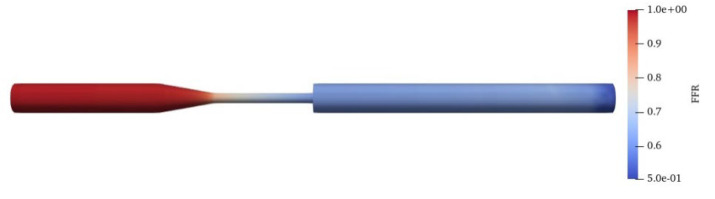
FFR distribution and shape of FDA nozzle model.

Additionally, the PBA approach was verified using the benchmark model of an FDA nozzle. The goal was to replicate the study by Stiehm et al. [18] using the PBA approach. The computational results for axial velocity along the centerline of the nozzle geometry were compared to the CFD data from the FDA’s round-robin study [18] for validation. The inlet conditions shown in Figure 7 and Figure 8 were the same as those in the Stiehm et al. [18] study. The FFR distribution along the profile of the FDA nozzle can be seen in Figure 22, but we used the velocity results along the centerline for validation.

**Figure 23:**
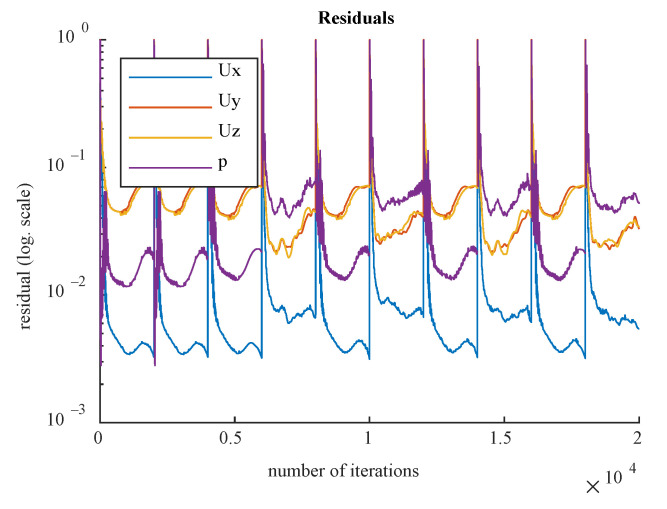
Residual history for steady-state simulation with shape of FDA nozzle model.

**Figure 24:**
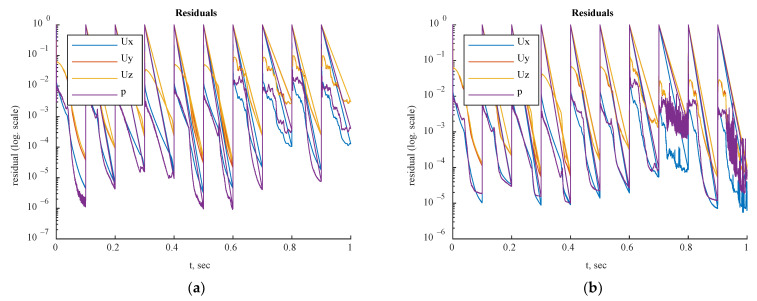
Residual histories for transient sinus (**a**) and waveform (**b**) with shape of FDA nozzle model.

Residual values for the steady-state and transient simulations are shown in Figure 23 and Figure 24, respectively. Based on the appropriate convergence of the residual values, the findings could be considered acceptable.

**Figure 25:**
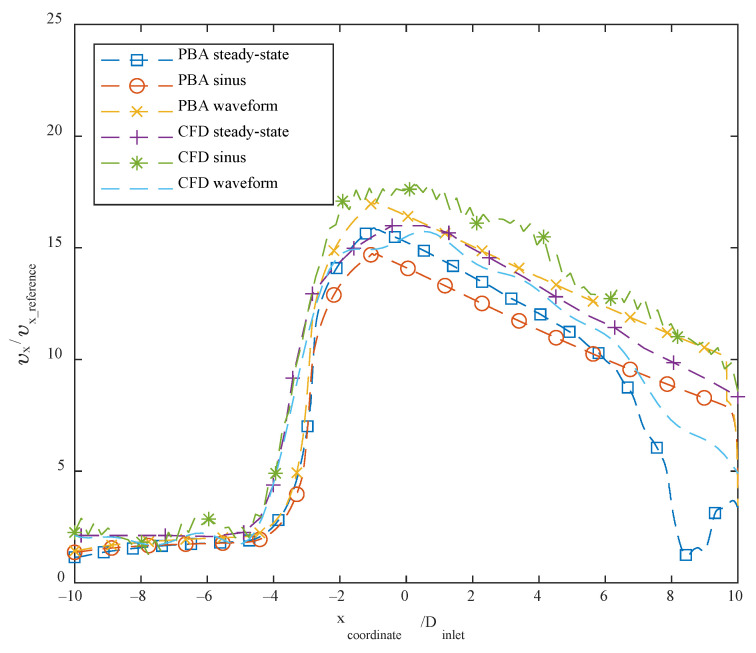
Axial flow rate along nozzle centerline: CFD results under steady-state and transient conditions and CFD PBA results under steady-state and transient conditions.

The axial velocity results for the CFD steady-state and CFDPBA databases for the idealized FDA nozzle matched closely, as shown in Figure 25. Additionally, the time-averaged axial velocity obtained from the transient CFD simulation with waveform inlet conditions agreed well with the steady-state PBA velocity data. However, there were minor differences downstream of the nozzle between 5D and 10D (Figure 7). The inclusion of wave pressure in the PBA algorithm caused the PBA sinus and waveform deviation. Since the pressure was constant in the steady-state example, the findings were not affected, but the waveform pressure caused a slight fluctuation in the centerline velocity values.

## 4. Conclusions

In this work, a physiologically based algorithm (PBA) was proposed and implemented in OpenFOAM CFD solver software to simulate blood flow in three coronary artery tree models and an FDA nozzle model. The performance of the proposed PBA was assessed using OpenFOAM and other CFD solvers [19]. The PBA, which was based on Murray’s law and patient-specific conditions at the inlet, could accurately calculate the outlet boundary conditions iteratively, a task that is difficult for both traditional LPM methods and invasive measurements. The PBA was found to work well with all the CFD solvers, such as Simvascular and OpenFOAM, and it could accurately predict FFR values in the cases tested. The results of the PBA were also compared with those of the LPM method, which was performed in ANSYS CFX [19]. The FFR values obtained from simulations using the suggested PBA were in excellent agreement with experimental ICA measurements. Unlike traditional methods, the PBA always guaranteed quick convergence in steady-state cases to an accurate solution for every situation. Because it is significantly more accessible and effective than the conventional Windkessel circuit analogy technique, this noninvasive FFR estimate using a PBA is a potential strategy for patient-specific detection of coronary stenosis. Our study further suggests that the suggested PBA, combined with an effective CFD solver, could be used as a low-cost, effective, and precise tool for diagnosing CAD. In individual patients, the PBA method has the potential to replace the expensive and risky invasive coronary angiography (ICA) procedure. Future research on pulsatile flows in CATs with fluid–structure interaction using the PBA is suggested.

We also used a nozzle model in this work that was based on the FDA’s benchmark geometry. By comparing the estimated axial velocity profile to CFD and CFDPBA data provided by the FDA, normalized fluid mechanical quantities could be used to further validate the method and its results.

Finally, two different transient CFD simulations were performed utilizing a sinus inlet condition and a physiological waveform. We concluded that, if only time-averaged results were examined, steady-state simulations were appropriate for hemodynamic studies. This approach may reduce the need for computing resources in further hemodynamic studies.

## Figures and Tables

**Table 1:** Workflow procedure of rapid iterative algorithm.

Timestep	Initial Conditions		Recorded	
	Inlet	Outlet	Inlet	Outlet
1st round of iterations	*p_in_* (table value) ^a^	Q *_i_	*Q_2i_*	*p_2i_*
2nd round of iterations	*Q_in_* (table value) ^b^	*p_2i_*	*p_3i_*	*Q_3i_*
3rd round of iterations	*p_in_* (table value)	*Q_3i_*	*Q_4i_*	*P_4i_*
…	…	…	…	…

*I*—number of coronary branches. * indicates calculated by Murray’s law at beginning. ^a^ Experimentally measured distal pressure at inlet of blood vessel. ^b^ Experimentally measured flow rate at inlet of blood vessel. ${Q_{in};P_{in};Q}_{i};P_{i}$—CFD-solver-calculated values of pressure and flow rate at inlets and outlets at different stages.

**Table 8:** Experimentally obtained input parameters for simulation of FDA nozzle [18].

Parameter	Value
Experimental inlet pressure $P_{exp}$	63.61 mm Hg (8480 Pa)
Experimental inlet flow rate $v_{exp}$	0.184 m/s

**Table 9:** Mesh information for CT209 model [19].

Mesh Size (mm)	Number of Cells	Number of Points
0.1702	3,949,528	674,346
0.2000	2,427,621	421,477
0.2400	1,400,809	248,645
0.2600	1,100,097	197,318

**Table 10:** Mesh information for CHN13 model [19].

Mesh Size (mm)	Number of Cells	Number of Points
0.22	1,166,473	211,638
0.35	286,803	57,015
0.53	80,093	18,104

**Table 11:** Relative errors calculated between the invasive and calculated FFRs using Simvascular, Ansys CFX, and OpenFOAM.

Model	Calculated FFR				Invasive FFR	Relative Error, %			
	Ansys CFX	Simvascular	OpenFOAM Steady-State PBA	OpenFOAM Transient PBA		Ansys CFX	Simvascular	OpenFOAM Steady-State PBA	OpenFOAM Transient PBA
CT209	0.753	0.758	0.762	0.80	0.76	0.92	0.26	0.26	5.26
CHN03	0.87	0.872	0.86	0.859	0.86	1.16	1.38	2.33	0.02
CHN13	0.658	0.691	0.683	0.69	0.68	3.24	1.59	0.44	1.47

## Data Availability

Upon request by emailing nursultan.alzhanov@nu.edu.kz.

## Appendix A

The Physiologically Based Algorithm, written in bash code, for steady-state computational fluid dynamics simulation.

## Appendix B

The Physiologically Based Algorithm, written in bash code, for transient computational fluid dynamics simulation.

## Appendix C. List of abbreviations

**Abbreviation**	**Definition**
CAD	Coronary artery disease
CAT	Coronary artery tree
CCTA	Coronary Computed Tomography Angiography
CFD	Computational fluid dynamics
CT	Computerized tomography
CTA	Computerized tomography angiography
FDA	U.S. Food and Drug Administration
FFR	Fractional flow reserve
FVM	Finite Volume Method
ICA	Invasive coronary angiography
IREC	Institutional Research Ethics Committee
LAD	Left Anterior Descending
LPM	Lumped parameter model
LPNM	Lumped Parameter Network Model
ODE	Ordinary Differential Equation
PBA	Physiologically based algorithm
